# Pulmonary high‐grade fetal adenocarcinoma associated with cystic airspace: A case report

**DOI:** 10.1111/1759-7714.13407

**Published:** 2020-03-29

**Authors:** Hironori Ishida, Masanori Yasuda, Hiroyuki Nitanda, Akitoshi Yanagihara, Ryo Taguchi, Ryuichi Yoshimura, Tetsuya Umesaki, Hirozo Sakaguchi, Yoshihiko Shimizu

**Affiliations:** ^1^ Department of General Thoracic Surgery Saitama Medical University International Medical Center Saitama Japan; ^2^ Department of Pathology Saitama Medical University International Medical Center Saitama Japan; ^3^ Department of Pathology Saitama Cardiovascular and Respiratory Center Saitama Japan

**Keywords:** Adenocarcinoma, cysts, fetal, lung, smoking

## Abstract

Lung cancers associated with cystic airspaces have a life‐threatening risk of a missed or delayed diagnosis. Here, we report a case of pulmonary high‐grade fetal adenocarcinoma, a rare lung carcinoma associated with cystic airspaces, as confirmed by computed tomography (CT) scan. A 73‐year‐old asymptomatic male with a 52‐pack a year smoking habit was referred to our hospital. Lung CT showed a thin‐walled cystic space with exophytic and endophytic solid nodules along the cyst wall. After surgery, histological analysis of a resected lung specimen revealed a pure high‐grade fetal adenocarcinoma probably associated with emphysematous bullae in pulmonary emphysema, suggesting smoking contributed to this pure form, as well as the emphysema. In conclusion, when treating elderly men with a smoking history, physicians need to carefully examine the walls of cystic airspaces on CT for fetal adenocarcinoma.

**Key points:**

**Significant findings of the study**

• Pulmonary high‐grade fetal adenocarcinoma may be associated with emphysematous bullae manifesting as cystic air spaces as shown by computed tomography.

**What this study adds**

• When scanning by computed tomography, physicians should carefully examine the pulmonary cystic airspace walls in elderly men with a smoking history.

## Introduction

Lung cancers associated with cystic airspaces have become more frequently recognized because of computed tomography (CT) scanning in clinical practice and lung cancer screening.^1^, ^2^ However, to date, a case of fetal adenocarcinoma of the lung has not been reported.

Fetal adenocarcinoma is rare, accounting for 0.4% of primary lung cancers, and is characterized by complex glandular structures resembling airway epithelium in the pseudoglandular phase of the fetal lung. Fetal adenocarcinomas are subcategorized into low‐ and high‐grade groups, the latter tending to occur in elderly male smokers, unlike the former which occurs in younger patients.^3^, ^4^ Most high‐grade fetal adenocarcinoma cases are histologically mixed. Few cases of the pure type, consisting entirely of fetal components, have been described.^5^

Here, we report a case of a pure high‐grade fetal adenocarcinoma of the lung associated with cystic airspaces on CT images in a 73‐year‐old man with a history of heavy smoking.

## Case report

**Figure 1:**
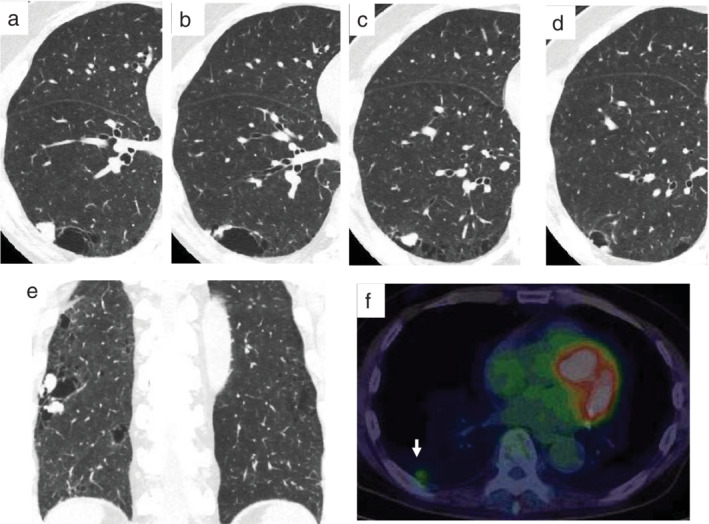
Diagnostic imaging. (**a**–**d**) Serial axial section images in a lung window setting on computed tomography showed a thin‐walled cystic airspace with exophytic and endophytic solid nodules along the cyst wall in the right lower lobe. (**e**) A coronal section image showed several multisided nodules abutting the wall of a multilocular cystic airspace in the emphysematous lung. (**f**) 18F‐FDG–positron emission tomography showed marginal FDG uptake, with a maximum standardized uptake value of 2.6 in the lung nodule (arrow). FDG, fluorodeoxyglucose.

A 73‐year‐old asymptomatic male, who had smoked 20 cigarettes per day from the age of 20 to 72 years and ceased a year prior to examination, was referred to our hospital for treatment of an abnormal pulmonary cystic space with nodules on CT. He had been undergoing treatment for hypertension, angina pectoris, and chronic kidney disease. Blood analysis did not reveal pulmonary infectious disease, including mycobacterial and fungal infections. A pulmonary function test revealed a forced expiratory volume in 1 second (FEV1) of 2.20 L and a reduced FEV1/forced vital capacity ratio (FEV1%) of 68% (<70%), suggesting a comorbidity of obstructive ventilatory disturbance due to smoking. Physical chest and abdominal examinations were unremarkable. Axial serial CT images in a lung window setting showed a thin‐walled cystic space with exophytic and endophytic solid nodules along the cyst wall. 18F‐fluorodeoxyglucose (FDG) positron emission tomography revealed marginal FDG uptake, with a maximum standardized uptake of 2.6 in the lung nodule (Fig 1), but excluded extrathoracic malignancies. These findings suggested a lung cancer associated with cystic spaces.

**Figure 2:**
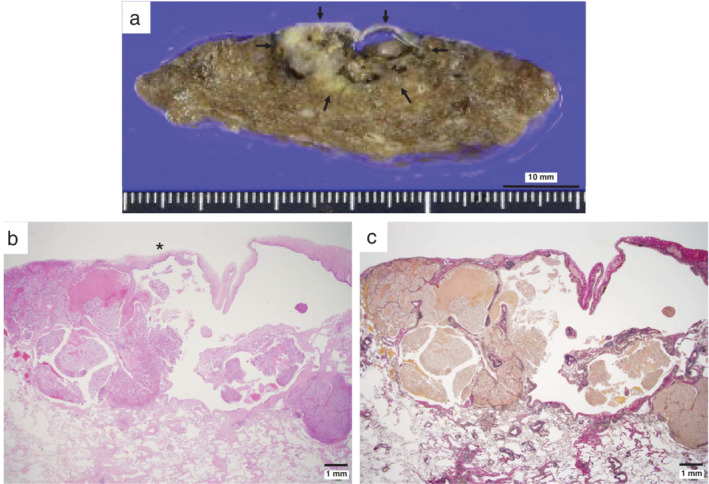
Macroscopic and histological findings. (**a**) A resected lung specimen revealed a tan‐whitish solid tumor with a thickened cyst wall (arrow), measuring 2.3 cm in length. The tumor showed focal necrosis. (**b** and **c**) Histological findings using hematoxylin and eosin (**b**), and elastic Verhoeff van Gieson stains (**c**) at a low magnification revealed an endophytic growing tumor in the septal walls and an exophytic tumor abutting the wall associated with a pre‐existing emphysematous bulla.

**Figure 3:**
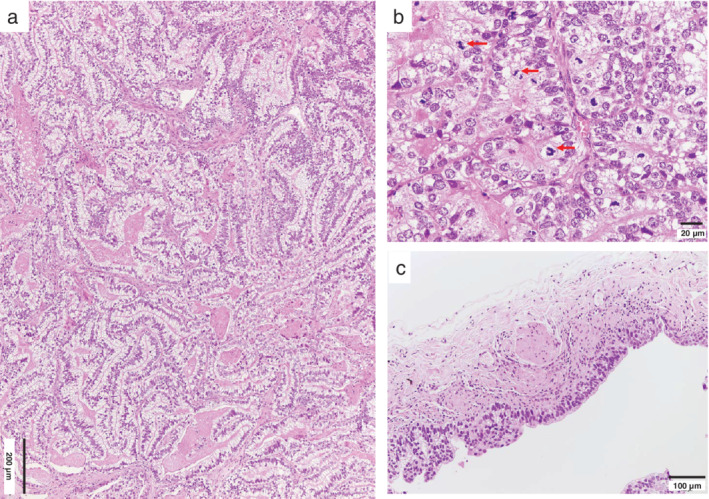
Microscopic finding. (**a**) Complex glandular, cribriform, and papillary structures are composed of clear columnar cells with pseudostratified nuclei and prominent nuclear atypia resembling fetal lung tubules. (**b**) Frequent mitotic figures are seen (arrows). (**c**) The inner surface of the wall near the tumor nodule is lined by tumor cells (see asterisk Fig 2b).

After wedge resection of the cyst with nodules, an adenocarcinoma was intraoperatively diagnosed. We subsequently performed a right lower lobectomy with lymph node dissection. Grossly, the sliced resected specimen revealed a tan‐whitish solid tumor with a cyst, 2.3 cm in length that showed walls of various thicknesses (Fig 2). Necrotic foci were visible. Histopathology revealed complex glandular structures comprising nonciliated atypical cells resembling developing epithelium in the pseudoglandular phase of the fetal lung (Fig 3a, b). Nuclear atypia with frequent mitotic figures was observed (Fig 3b). The inner wall surfaces showed a lining of tumor cells (Fig 3c), as well as squamous metaplasia or bronchiolization.

**Figure 4:**
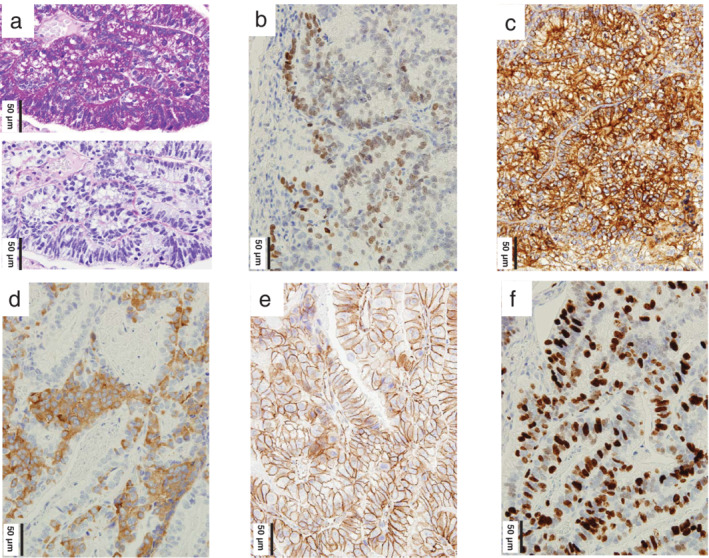
Special and immunohistochemical stains. (**a**) Positive periodic acid‐Schiff (PAS; top) and negative PAS‐D (bottom) stains in combination with diastase, an enzyme that breaks down glycogen, are shown. Abundant glycogen in the cytoplasm of the tumor cells is evident. (**b**–**d**) Tumor cells are positive for (**b**) Sal‐like protein 4 (SALL4), (**c**) α‐fetoprotein, and (**d**) synaptophysin. (**e**) β‐catenin is expressed only in the tumor cell membrane. (**f**) Ki‐67 staining shows a high labeling index of 55%.

Positive periodic acid‐Schiff (PAS) and negative PAS‐diastase stains revealed glycogen‐rich tumor cells (Fig 4a). Immunohistochemically, tumor cells were positive for oncofetal (SALL4 and α‐fetoprotein), neuroendocrine (synaptophysin, CD56 [not shown], and chromogranin A [not shown]) markers. β‐catenin was expressed only in the tumor cell membrane (Fig 4b–e). Tumor cells were negative for thyroid transcription factor 1 and programmed cell death ligand 1 (PD‐L1). Ki‐67 staining revealed increased cell proliferation (Fig 4f). Histological analysis of the entire tumor did not reveal any conventional‐type lung adenocarcinoma (acinar, papillary, and lepidic), large cell neuroendocrine carcinoma, sarcomatoid component or morula. Resected lymph nodes did not show metastases. Background lung tissue revealed pulmonary emphysema.

A pure high‐grade fetal adenocarcinoma staged at pT1cN0M0 (stage IA3) was diagnosed.^5^ Epidermal growth factor receptor (*EGFR*) mutations or anaplastic lymphoma kinase (*ALK*) rearrangements were not found in the tumor. A total of 12 months after surgery, the patient's cancer had not recurred.

## Discussion

High‐grade fetal adenocarcinoma may be associated with cystic airspaces visible on CT scan. Smoking may contribute to this tumor, as well as emphysematous bullae manifesting as cystic airspaces. Here, we report the first case of a pure pulmonary fetal adenocarcinoma associated with cystic airspaces.

Lung cancers associated with cystic airspaces have a risk of missed or delayed diagnoses. “Cystic airspace” broadly encompasses emphysematous bulla, congenital or fibrotic cysts, subpleural bleb, bronchiectasis, and cystic dilatation by distal airway obstruction, each associated with lung cancer.^6^, ^7^ Cystic airspaces may precede cancers and vice versa. The frequency of such cases among lung cancer patients is 1.0%–3.7%, with the majority being former and current smokers with pulmonary emphysema.^6^ This may explain why cysts interfere with ventilation and lung clearance and thus facilitate carcinogen deposition.^2^ However, the exact carcinogenic mechanism remains unclear. The most common histological lung cancers associated with cystic airspaces are conventional adenocarcinomas such as lepidic, papillary, or acinar adenocarcinomas, followed by squamous cell carcinomas.^1^, ^2^ Notably, fetal adenocarcinoma has not been reported, although data regarding adenocarcinoma subtypes is limited.

Fetal adenocarcinoma presents as complex glandular structures resembling fetal lung tubules. Unlike low‐grade fetal adenocarcinoma, the high‐grade form shows prominent nuclear atypia, necrosis, and no morula. This high‐grade form coexists frequently with conventional adenocarcinomas, and occasionally with large cell neuroendocrine carcinomas and enteric adenocarcinomas.^5^ Immunohistochemically, the fetal component is often positive for oncofetal and neuroendocrine markers. High‐grade fetal adenocarcinoma is defined as a diagnosis when >50% fetal morphology is present according to the 2015 World Health Organization classification.^8^, ^9^ Furthermore, high‐grade fetal adenocarcinomas consist of pure (entirely fetal morphology) or mixed forms. To date, only seven cases of pure high‐grade fetal adenocarcinoma, including the present case, have been described, all in elderly men with a heavy smoking history.^5^, ^10^, ^11^ In addition, none of the seven pure form cases (including the present case) harbored *EGFR* mutations and *ALK* rearrangements.^5^, ^10^, ^11^ A prior case that was tested for PDL‐1 did not show any expression in addition to the present case.^11^

High‐grade fetal adenocarcinomas represent aggressive biological behavior.^9^ Although cigarette smoke genetically contributes to dysregulation of cell‐cycle and tumor progression,^12^ distinctive molecular features of the pure form remain to be investigated.^5^ A recent study suggested a high‐grade fetal adenocarcinoma component (≥5%) in lung adenocarcinomas is significantly associated with a poor prognosis.^9^ All six patients with pure high‐grade fetal adenocarcinomas had stage IIB–IV disease after surgery, most experiencing recurrence within two years^5^, ^10^, ^11^; however, post‐surgically, the present case was of the lowest level, stage IA.

Previous CT images were unavailable; however, the cystic airspaces may have preceded the cancer, given a history of bullous emphysema. A total of 10 patients (19%) with high‐grade fetal adenocarcinoma showed severe pulmonary emphysematous changes.^9^ As solid components increase with time, cystic airspaces (eg, emphysematous bullae) may gradually decrease in size, finally giving way to a completely solid lesion. Therefore, the early detection of such fetal adenocarcinomas associated with cystic airspaces may contribute to improved outcomes.

In conclusion, pure high‐grade fetal adenocarcinoma of the lung may be associated with emphysematous bullae presenting as cystic airspaces on CT, suggesting smoking is involved in such cancers. Physicians should pay careful attention to the walls of cystic airspaces, as viewed on CT, in elderly men who smoke heavily.

## Disclosure

The authors report no conflicts of interest.
